# Examining equity in access and utilization of a freely available meditation app

**DOI:** 10.1038/s44184-023-00025-y

**Published:** 2023-04-18

**Authors:** Zishan Jiwani, Raquel Tatar, Cortland J. Dahl, Christine D. Wilson-Mendenhall, Matthew J. Hirshberg, Richard J. Davidson, Simon B. Goldberg

**Affiliations:** 1 Department of Counseling Psychology, University of Wisconsin, Madison, WI USA; 2 Center for Healthy Minds, University of Wisconsin, Madison, WI USA; 3 Healthy Minds Innovations, Madison, WI USA

**Keywords:** Health care, Health services, Public health, Quality of life

## Abstract

Digital interventions have the potential to alleviate mental health disparities for marginalized and minoritized communities. The current study examined whether disparities in access and utilization of meditation in the United States (US) were reduced for a freely available meditation app. We analyzed demographic and usage data from US-based users of the Healthy Minds Program (HMP; *N* = 66,482) between October 2019 and July 2022. College education was associated with a greater likelihood of accessing (65.0% of users vs. 32.9% of the US population) and continuing to utilize the app (*β* = 0.11–0.17). Conversely, identifying as African American was associated lower likelihood of accessing (5.3% vs. 13.4% of the US population) and continuing to utilize the app (*β* = −0.02–0.03). African Americans were more likely to access content from an African American meditation teacher, but this did not appear to increase utilization. Additional efforts are warranted to identify factors that might reduce disparities.

## Introduction

Smartphones have become increasingly common in daily life for most people in the United States (US), with ~85% of Americans owning some type of smartphone^1^. Given the widespread use of smartphones, it has been suggested that digital technology may play a critical role in reducing disparities in healthcare access for marginalized and minoritized communities^2,3^. Within the context of mental health, digital technology has the potential to alleviate three key barriers which have been found to reduce service utilization for marginalized populations: financial burden, ease of accessibility for hard-to-reach populations, and stigma associated with the use of mental health services^4^. Additionally, the use of digital technology to deliver mental health care may be particularly relevant for marginalized communities. For instance, compared with non-Latinx (NL) White individuals, Latinx and African American individuals are more likely to rely on smartphones to access the internet^5^. Lower-income and younger individuals have also been found to use smartphones to access health information more commonly than higher-income and older individuals^6^. That said, certain groups, such as older, less educated, and low-income individuals are at once less likely to own smartphones than younger, college-educated and high-income individuals^1^.

Amongst consumer-facing smartphone applications (apps), behavioral health apps comprise nearly one-third of the global market for health and wellness apps^7^. Most downloaded behavioral health apps have been found to focus on meditation, mindfulness, or relaxation^8^. Additionally, a recent study found that amongst behavioral health apps in 2018, two mindfulness apps (Calm and Headspace) were responsible for 90% of monthly active users^9^. The popularity of meditation-based apps suggests that such apps may be an important tool in improving mental health and reducing disparities in access to mental health care.

Mindfulness and meditation practice has become popular in the US. In 2017, nearly one in five American adults practiced some type of meditation^10^. Meditation-based interventions (MBIs) have also been found in randomized clinical trials (RCTs) to produce benefits for a wide range of psychological disorders^11,12^ and meditation-based apps have also shown promise in improving mental health^13^. Additionally, recent meta-analyses have shown that MBIs may have beneficial effects on vulnerable populations such as people of color^14^. With appropriate adaptations, MBIs have the potential to benefit marginalized groups by helping cultivate emotion regulation, compassion, and awareness skills which may help manage environmental and race-based stressors^15–19^. Nonetheless, results from National Health Interview Survey (NHIS) indicate that in the US, well-educated, NL White and female-identifying individuals are more likely to engage in meditation practices relative to lesser educated, non-White, and male groups^10,20^. Thus, while MBIs may be an effective tool to reduce psychological distress for vulnerable groups, current evidence suggests that it has been less utilized by these groups.

In the context of meditation-based apps, recent studies with large samples have found a similar trend. Huberty and colleagues^21^ examined utilization patterns amongst 12,151 paying subscribers to the Calm app and found that the sample was predominantly female (79.94%) and NL White (81.41%). Comparably, Bhuiyan and colleagues^22^ examined 8,392 Calm users and found that the sample was mostly female (83.9%), NL White (91.8%), and college-educated (86.4%). However, one important potential barrier to access and utilization of meditation apps such as Calm is their cost which may limit accessibility^23^. A recent scoping review of mental health app evaluation frameworks by Ramos and colleagues^24^ found that cost of access was the most frequently included diversity, equity, and inclusion (DEI) criteria across frameworks. Thus, app developers and evaluators consider cost as a central factor in increasing utilization. Conversely, Ramos and colleagues^24^ also found that diverse representation was only considered by one evaluation framework. Diverse representation (i.e., appearance and names of individuals portrayed in the app) and diversity in the persons providing care (i.e., appearance and names of individuals who are portrayed to be providing the intervention) are considered to be important components of cultural adaptation of mobile-based interventions^25^. In psychotherapy research, racial and ethnic minoritized individuals, particularly African Americans, have been found to prefer therapists who matched their racial or ethnic identity^26,27^. Such preferences may extend to meditation apps as well, although this has not been established to date. Kozlov and colleagues^28^ examined utilization in a freely available meditation app with a large sample (*N* = 104,067) but did not report or examine demographic variability in utilization. As such, it remains unclear whether a freely available meditation app might be able to reduce disparities in meditation access and utilization in the general population in the US and if factors such as the diversity of persons delivering content might influence app utilization.

The current study had three aims. First, we examined the demographic distribution of US-based users of the Healthy Minds Program (HMP), a meditation-based well-being app freely available on Apple and Google app stores, relative to the general population in the US. Second, for users who have accessed the app, we assessed if demographic characteristics predict the utilization and practice of the app. Finally, the app allows participants to select from four different meditation practice speakers with varying racial/ethnic identities to guide their practice. We assessed if user demographics, particularly racial and ethnic identity, predicted utilization of practice speakers with diverse identities and as exploratory analyses, whether utilization of speakers of diverse identities was associated with greater utilization of the app.

## Method

### Participants and procedure

Data for the study was drawn from mobile analytics of public use data of the HMP between September 2019 and July 2022. HMP is a meditation-based app freely available on Apple and Google app stores that is based on four pillars of well-being proposed by Dahl and colleagues^29^ which include awareness, connection, insight, and purpose. HMP contains modules focused on each of these pillars that include a combination of evidence-based didactics and guided meditation practice sessions designed to cultivate each aspect of well-being. For the guided practices, users can choose to practice between 5 and 30 min and select from one of four speakers with varying genders and racial/ethnic identities whose names and photos are displayed in the app along with a link to get additional information. The spoken content of the guided practice is similar across speakers. An NL White male who was central in the development of the HMP app is featured most prominently throughout and is the default choice to guide meditation practice sessions. Based on user experience feedback suggesting a need to increase representation in the app, a speaker who identifies as Latinx female was initially added as a meditation practice speaker option, and in subsequent updates of the app, additional speakers who identified as Asian male and African American female were added to the app. However, the introduction and all didactic content are still led by the NL White male speaker. The HMP has been found to be effective in reducing psychological distress and improving measures of well-being and social connectedness relative to a waitlist control across two RCTs^30,31^.

We initially identified 105,266 unique HMP users who had downloaded the app from a US Apple or Google app store between September 2019 and July 2022. Participants (*N* = 66,482, 63.2%) were included if they reported at least one demographic variable. We limited our examination to the first 30 days of participant utilization of the app. Additionally, all users who accessed the app less than 30 days prior to the last day of the examination were excluded. As part of the HMP terms and conditions, participants indicate their consent to the use of deidentified data for research purposes. Study procedures were approved by the Institutional Review Board at the University of Wisconsin-Madison with a waiver of informed consent.

### Measures

We defined access to the HMP app as having downloaded the app. As described in the Analytic Strategy section, we compared the demographics of those accessing the HMP app with the US Census.

Utilization of HMP was operationalized in three different ways. *Sum days* were the total number of days a participant utilized the app within the 30 days of accessing the app. Utilization could include meditation practice, didactic instruction, or responding to questions about well-being. *Sum practice* reflected the total number of meditation practice sessions a participant completed. *Any practice* was a dichotomous variable created to examine if the participant utilized any meditation practices after downloading the app with 1 indicating at least one meditation practice session and 0 indicating no practice sessions. Practice variables (i.e., sum practice and any practice) do not include didactic components of the app. Assessments of utilization excluded the use of the app to complete the demographic survey.

To assess how frequently participants utilized various meditation practice speakers, proportions for each speaker were operationalized using the number of sessions with a particular speaker divided by the total practice sessions with any of the four speakers. To calculate speaker proportions, participant utilization of unguided meditation sessions or specialized meditation sessions outside the five core modules was excluded.

Five demographic variables were included in the study: age, ethnicity/race, gender, education, and relationship status. Demographic variables were collected in the app categorically. Age was collected categorically in the app, and the categories shifted over time (e.g., the 19–24 category was later changed to 18–24). As age 34 was a consistent cut point in the categories assessed in the current study, has been used as a cut point for a younger age category in prior epidemiologic work investigating access to mental health treatment^32^, we dichotomized age into 34 and younger or 35 and older. The race/ethnicity variable was adapted following the structure proposed by the US Census Bureau (2022). Six racial-ethnic groups were included: (1) NL White, (2) Black or African American, (3) Hispanic or Latinx, (4) Asian and (5) Native American, Hawaiian or Other Pacific Islander, and (6) Other (i.e., “none of the above fully describe me”). The Census also includes data on individuals who identify as multiracial, but this category was not available as an option for app users and thus was not included in the sample. Gender was dichotomized to individuals who identified as female and all other groups. We examined whether utilization differed as an outcome of identifying as male versus identifying as a third gender category which was a combination of participants identifying as either non-binary or something else. We did not see a difference and thereby collapsed the two groups in the analyses for the purpose of keeping demographic variables consistent across analyses. Education was dichotomized between those with a college degree and those without a college degree. Finally, marital status was dichotomized between those currently married or in a domestic partnership and those who did not describe themselves as either married or in a domestic partnership.

### Analytic strategy

Data were analyzed in R^33^. To examine access, demographic proportions of app users who downloaded the app and provided demographic information were compared with population proportions from the most recent US Census^34^ using one proportion *z* tests. Utilization was examined using linear multiple regression models for continuous outcome variables (sum days and sum practice) and logistic multiple regression models for the dichotomous outcome variable (any practice) with demographic variables included as predictors. Identification as NL White was used as the reference group for the race/ethnicity variable with the non-NL White group dummy coded. Non-college-educated, age greater than 34, non-female identification, and unmarried/divorced were used as reference groups for the remaining demographic variables. Given the wide variability in utilization, sum days and sum practice were winsorized to two standard deviations^35^. Models were run with the data available (i.e., pairwise deletion). Standardized betas were calculated using the ‘lm.beta’ function^36^ and odds ratios were calculated for logistic regression.

To assess utilization with meditation practice speakers of diverse identities, we examined whether demographic factors were associated with the practice speaker proportions for each speaker in multiple regression models with all demographic variables included. Given two speakers (Asian Male and African American female) were added to the app later than the initial two speakers (NL White Male and Latinx Female), these analyses were limited to the period after all four speakers were included and participants utilized at least one meditation practice (*n* = 10,737). In exploratory analyses, we assessed if greater utilization of diverse practice speakers was associated with greater app utilization overall. To do this, we examined whether the association between demographic variables and the number of meditation practice sessions (i.e., sum practice) was moderated by practice speaker proportion for each of the practice speakers in multiple regression models. Practice speaker options were not provided for the didactic content and thus the number of days of app utilization, which includes didactic content, was not included as a variable in this analysis.

To control for Type I error rates, a Bonferroni^37^ correction procedure was utilized. When examining associations between demographics and the three metrics of utilization, α was set to 0.017 (0.05/3) and when examining associations between demographics and the four-speaker proportions, including for exploratory analyses, α was set to 0.013 (0.05/4). Guidelines suggested by Cohen^38^ were followed to interpret the magnitude of effect sizes for continuous variables and guidelines suggested by Chen and colleagues^39^ for the interpretation of odds ratios.

We conducted several sensitivity analyses to check the robustness of the results for the associations between the sum practice variable and practice speaker proportions with demographics. First, we examined if the results were consistent when restricting utilization to the first seven rather than the first 30 days after downloading the app. Second, for the association between utilization and demographics, we examined if the results for the continuous variables (sum practice and sum days) were consistent without winsorizing. Third, for associations between practice speaker proportions and demographics, we assessed if the results were consistent if we restricted the sample to participants with two or more meditation practices in the core modules. This restriction was examined as two practices would allow participants the opportunity to utilize more than one meditation practice speaker.

## Results

### Access

Access was analyzed by comparing the proportions of US-based HMP app users with population proportions from the US census (Table 1). Nearly two times as many HMP users reported being college educated relative to the general US population (65.0% vs. 32.9%, for the HMP user sample and the US population, respectively, *p* < 0.001). Additionally, significantly larger proportions of HMP users identified as female (69.8% vs. 50.8%, *p* < 0.001), NL White (76.7% vs. 60.1%, *p* < 0.001), and between 18–34 years of age (33.8% vs. 22.9%, *p* < 0.001) relative to the general US population. Conversely, a significantly smaller proportion of HMP users identified as African American (5.3% vs. 13.4%, *p* < 0.001), Latinx (7.8% vs. 18.5%, *p* < 0.001), and married (48.4% vs. 51.1%, *p* < 0.001). No significant differences were found between the proportion of HMP users identifying as Asian American (6.0% vs. 5.9%, *p* = 0.258) or as Native American or Pacific Islander (1.2% vs. 1.3%, *p* = 0.094) and the general US population.

**Table 1:** Proportion of HMP users versus US census proportions.

Variable	*N*	Prop (%)	US census (%)	*χ*^2^	*p*
Female	45,900	69.8	50.8	9464.64	<0.001
Age (18–34)	22,061	33.8	22.9	4383.86	<0.001
College	36,079	65.0	32.9	25892.47	<0.001
Married or domestic partnership	31,234	48.4	51.1	185.9	<0.001
Race and Ethnicity					
NL White	43,381	76.7	60.1	6482.92	<0.001
African American	2997	5.3	13.4	3200.18	<0.001
Latinx	4390	7.8	18.5	4327.95	<0.001
Asian	3385	6.0	5.9	0.69	0.407
Native American or Pacific Islander	694	1.2	1.3	2.31	0.128
Other	1729	3.1	NA	NA	NA

*HMP* Healthy Minds Program app, *Prop* proportion, *NL White* Non-Latinx White. *χ*^2^ based on tests with one degree of freedom. Statistical Test: one proportion Z test.

### Utilization

Descriptive statistics for the raw and winsorized HMP utilization variables are reported in Table 2. On average, participants utilized the app for 4.24 days (*SD* = 5.58) with a median utilization of two days within the 30 days following after accessing the app. A slight majority of participants (54.2%) utilized at least one meditation practice in the app. The average participant utilized meditation practices in the app for 3.88 sessions (*SD* = 8.06) with a median of one practice session and a range of 0 to 214 sessions.

**Table 2:** Descriptive statistics for HMP utilization variables.

Utilization variable	*Mean*	*SD*	Median	Min	Max	Range	Skew	Kurtosis	*SE*
Any Practice	0.54	0.50	1.00	0.00	1.00	1.00	−0.15	−1.98	0.00
Sum Days (2_wins)	3.85	4.34	2.00	1.00	15.40	14.40	1.65	1.49	0.02
Sum Days (raw)	4.24	5.58	2.00	1.00	30.00	29.00	2.37	5.54	0.02
Sum Practice (2_wins)	3.34	5.38	1.00	0.00	20.00	20.00	1.92	2.73	0.02
Sum Practice (raw)	3.88	8.06	1.00	0.00	214.00	214.00	5.21	50.12	0.03

*HMP* Healthy Minds Program app, *Any Practice* completion of any guided meditation practice, *Sum Days* number of days of app utilization, *Sum Practice* number of meditation practice sessions, *2 wins* winsorized to two standard deviations, *raw* variables without adjustment. *n* = 66,482.

Associations between demographics and utilization variables (sum days, sum practice, any practice) were assessed using multiple linear regression and logistic regression. Standardized betas (*β)*, odds ratio (*OR*), and p-values are presented in Table 3 for the continuous variables and Table 4 for the dichotomous practice variable. Identifying as college-educated was associated positively with number of days of app utilization (i.e., sum days, *β* = 0.17, *p* < 0.001) and number of meditation practice sessions (i.e., sum practice, *β* = 0.14, *p* < 0.001) as well as with greater likelihood of engaging in any meditation practice (i.e., any practice, *OR* = 1.77, *p* < 0.001). Identifying as being married or in a domestic partnership was also associated positively with the number of days of app utilization (*β* = 0.03, *p* < 0.001) and the number of meditation practice sessions (*β* = 0.01, *p* = 0.003) as well as with a greater likelihood of utilizing any meditation practice (*OR* = 1.05, *p* = 0.011), albeit with very small effect sizes.

**Table 3:** Standardized regression coefficients for associations between demographic variables and continuous HMP utilization variables.

		First 30 Days of HMP use				First 7 Days of HMP use			
Demographic variable	Utilization variable	*β*	95% CI for *β*		*p*	*β*	95% CI for *β*		*p*
			*LL*	*UL*			*LL*	*UL*	
Age (18–34)	Sum Days (2_wins)	0.00	−0.01	0.01	0.742	0.00	−0.01	0.01	0.927
Age (18–34)	Sum Days (raw)	−0.01	−0.02	0.00	0.067	0.00	−0.01	0.01	0.847
Age (18–34)	Sum Practice (2_wins)	−0.02	−0.03	−0.01	<0.001	−0.03	−0.04	−0.02	<0.001
Age (18–34)	Sum Practice (raw)	−0.03	−0.04	−0.02	<0.001	−0.04	−0.05	−0.03	<0.001
College	Sum Days (2_wins)	0.17	0.16	0.18	<0.001	0.15	0.14	0.16	<0.001
College	Sum Days (raw)	0.15	0.15	0.16	<0.001	0.15	0.14	0.16	<0.001
College	Sum Practice (2_wins)	0.14	0.13	0.15	<0.001	0.11	0.11	0.12	<0.001
College	Sum Practice (raw)	0.11	0.10	0.12	<0.001	0.09	0.08	0.10	<0.001
Female	Sum Days (2_wins)	−0.06	−0.07	−0.06	<0.001	−0.06	−0.07	−0.05	<0.001
Female	Sum Days (raw)	−0.07	−0.07	−0.06	<0.001	−0.06	−0.07	−0.05	<0.001
Female	Sum Practice (2_wins)	−0.06	−0.07	−0.05	<0.001	−0.05	−0.06	−0.04	<0.001
Female	Sum Practice (raw)	−0.05	−0.06	−0.05	<0.001	−0.04	−0.05	−0.04	<0.001
Married or domestic partnership	Sum Days (2_wins)	0.03	0.02	0.03	<0.001	0.02	0.01	0.03	<0.001
Married or domestic partnership	Sum Days (raw)	0.02	0.01	0.03	<0.001	0.02	0.01	0.03	<0.001
Married or domestic partnership	Sum Practice (2_wins)	0.01	0.01	0.02	0.003	0.01	0.00	0.02	0.098
Married or domestic partnership	Sum Practice (raw)	0.01	0.00	0.01	0.294	0.00	−0.01	0.01	0.713
Race African American	Sum Days (2_wins)	−0.03	−0.04	−0.02	<0.001	−0.03	−0.04	−0.02	<0.001
Race African American	Sum Days (raw)	−0.03	−0.04	−0.02	<0.001	−0.03	−0.04	−0.02	<0.001
Race African American	Sum Practice (2_wins)	−0.03	−0.03	−0.02	<0.001	−0.02	−0.03	−0.01	<0.001
Race African American	Sum Practice (raw)	−0.02	−0.02	−0.01	<0.001	−0.01	−0.02	0.00	0.016
Race Asian	Sum Days (2_wins)	−0.01	−0.02	0.00	0.024	−0.01	−0.02	0.00	0.047
Race Asian	Sum Days (raw)	−0.01	−0.02	0.00	0.112	−0.01	−0.02	0.00	0.068
Race Asian	Sum Practice (2_wins)	0.00	−0.01	0.01	0.956	0.00	−0.01	0.01	0.397
Race Asian	Sum Practice (raw)	0.01	0.00	0.02	0.008	0.01	0.01	0.02	0.002
Race Latinx	Sum Days (2_wins)	−0.01	−0.01	0.00	0.296	−0.01	−0.01	0.00	0.193
Race Latinx	Sum Days (raw)	−0.01	−0.01	0.00	0.266	−0.01	−0.02	0.00	0.155
Race Latinx	Sum Practice (2_wins)	0.00	−0.01	0.01	0.364	0.01	0.00	0.01	0.296
Race Latinx	Sum Practice (raw)	0.01	0.00	0.02	0.081	0.01	0.00	0.02	0.055
Race Native American or Pacific Islander	Sum Days (2_wins)	−0.02	−0.03	−0.01	<0.001	−0.02	−0.03	−0.01	<0.001
Race Native American or Pacific Islander	Sum Days (raw)	−0.02	−0.03	−0.01	<0.001	−0.02	−0.03	−0.01	<0.001
Race Native American or Pacific Islander	Sum Practice (2_wins)	−0.02	−0.03	−0.01	<0.001	−0.02	−0.02	−0.01	0.001
Race Native American or Pacific Islander	Sum Practice (raw)	−0.01	−0.02	−0.01	0.002	−0.01	−0.02	0.00	0.017
Race other	Sum Days (2_wins)	−0.01	−0.02	0.00	0.034	−0.01	−0.02	0.00	0.032
Race other	Sum Days (raw)	−0.01	−0.02	0.00	0.035	−0.01	−0.02	0.00	0.041
Race other	Sum Practice (2_wins)	−0.01	−0.01	0.00	0.286	0.00	−0.01	0.01	0.541
Race other	Sum Practice (raw)	0.00	−0.01	0.01	0.475	0.00	−0.01	0.01	0.820

*HMP* Healthy Minds Program app; *β* standardized beta, *CI* confidence interval, *LL* lower limit, *UL* upper limit, *Sum Days* number of days of app utilization, *Sum Practice* number of meditation practice sessions, *raw* variable without adjustment, *2_wins* winsorized to two standard deviations, *Age* (<34) Non-college educated, Non-females, unmarried/divorced and NL White used as the reference group. *n* = 66,482. Statistical test: multiple regression.

**Table 4:** Associations between demographic variables and likelihood of engaging in any meditation practice.

	First 30 days of HMP Use		First 7 days of HMP Use	
Demographic variable	*OR*	*p*	*OR*	*p*
Age (18–34)	0.97	0.136	0.95	0.010
College	1.77	<0.001	1.62	<0.001
Female	0.85	<0.001	0.87	<0.001
Married or domestic partnership	1.05	0.011	1.03	0.151
Race African American	0.94	0.091	0.94	0.080
Race Asian	0.85	<0.001	0.87	0.001
Race Latinx	1.05	0.179	1.02	0.574
Race Native American or Pacific Islander	0.68	<0.001	0.76	0.001
Race other	1.01	0.856	0.98	0.710

*OR* Odds ratio, *Age* (<34) non-college-educated, unmarried/divorced, and NL White were used as the reference group. *n* = 66,482. Statistical test: multiple regression.

**Fig. 1:**
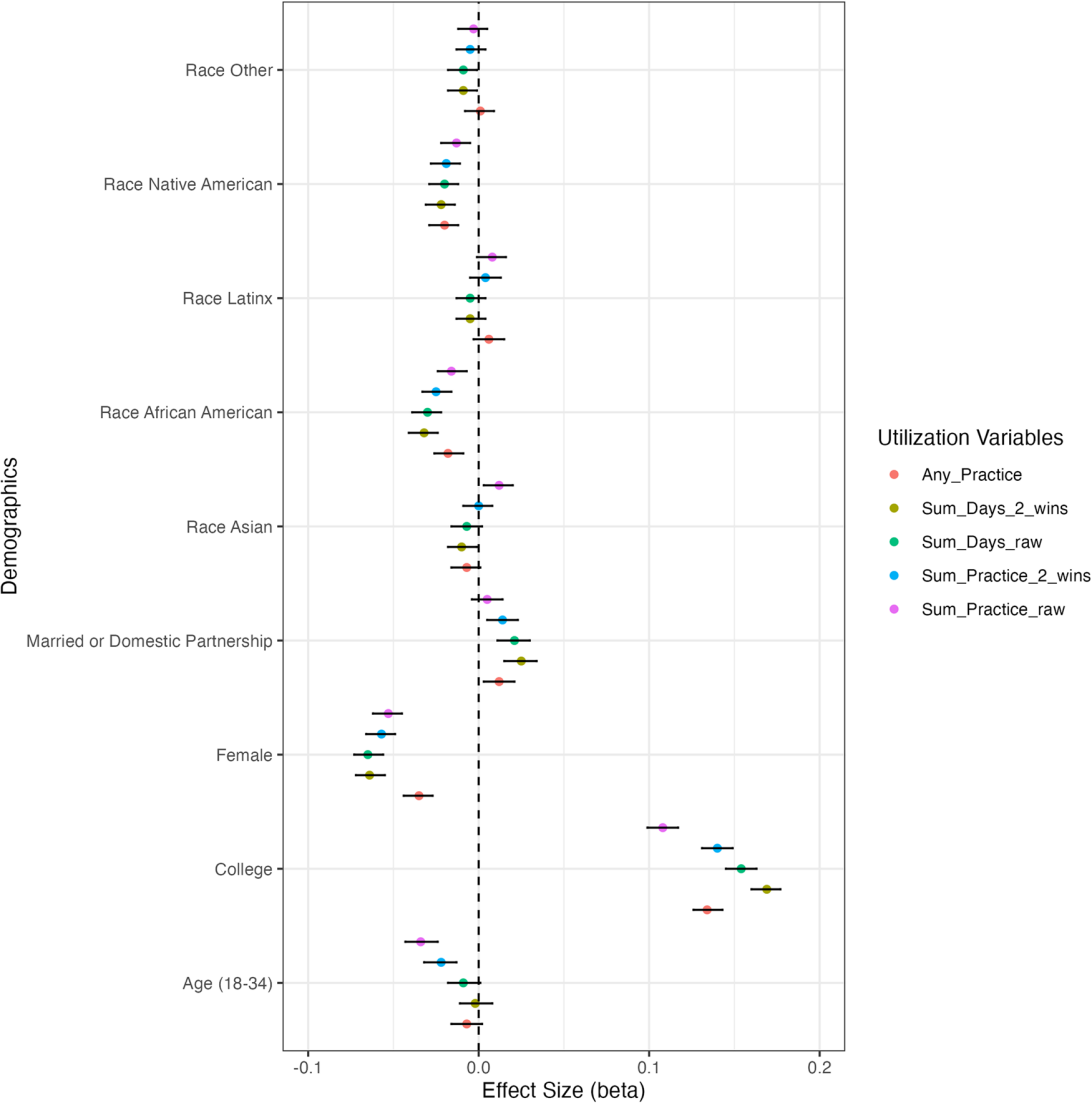
Associations between demographics and HMP utilization. HMP Healthy Minds Program, Race Native American Race Native American and Pacific Islander; *Age* (<34), non-college-educated, unmarried/divorced, and NL White used as the reference group. Statistical test: multiple regression. Error bars represent the 95% confidence interval.

Conversely, identifying as female was negatively associated with number of days of app utilization (*β* = −0.06, *p* < 0.001) and number of meditation practice sessions (*β* = −0.06, *p* < 0.001) as well as with lower likelihood of utilizing any meditation practice (*OR* = 0.85, *p* < 0.001). Additionally, identifying as African American or Native American/Pacific Islander was also associated with lower utilization relative to NL White participants. Specifically, identifying as African American was associated with lower number of days of app utilization (*β* = −0.03, *p* < 0.001) and number of meditation practice sessions (*β* = −0.03, *p* < 0.001) as well as with lower likelihood of utilizing any meditation practice (*OR* = 0.85, *p* < 0.001). Similarly, identifying as Native American was also associated with lower number of days of app utilization (*β* = −0.02, *p* < 0.001) and number of meditation practice sessions (*β* = −0.02, *p* < 0.001) as well as with lower likelihood of engaging in any meditation practice (*OR* = 0.68, *p* < 0.001). Identifying as a young person (18–34 years of age) was associated with fewer meditation practice sessions (*β* = −0.02, *p* < 0.001) but was not significantly associated with number of days of app utilization (*β* = −0.00, *p* = 0.728) or likelihood of engaging in any meditation practice (*OR* = 0.97, *p* = 0.136). Identifying as Asian, Latinx, or Other race was not significantly associated with any utilization variables. See Fig. 1 for a visual representation of the results.

As a sensitivity analysis, we examined whether associations with utilization were consistent when limiting our examination to the first seven days of app utilization as opposed to the first 30 days (Tables 3 and 4). Two changes in statistical significance did emerge. Being married was no longer significantly associated with the number of meditation practice sessions (*β* = 0.01, *p* = 0.098). Identifying as young was associated with a lower likelihood of engaging in any practice (18–34; *OR* = 0.95, *p* = 0.010).

Additionally, we examined if results varied when raw practice variables were used for the two continuous variables relative to winsorized continuous practice variables (Table 3). Two changes in statistical significance did emerge. Specifically, the association between being married and the number of meditation practice sessions was no longer significant. Additionally, a small positive association emerged between identifying as Asian and the raw number of meditation practice sessions (*β* = 0.01, *p* = 0.008). See Fig. 1 for a visual representation of the results.

### Practice speaker utilization

Results of multiple regression models examining associations between demographics and proportion of utilization for each of the four speakers available in the app are presented in Table 5. Examining the variation across race, relative to NL White individuals, identifying as African American was associated with higher utilization of the Black female speaker (*β* = 0.06, *p* < 0.001). Relative to NL White identifying individuals, identifying as Asian was positively associated with higher utilization of the NL White male speaker (*β* = 0.03, *p* = 0.012) and lower utilization of the Black female speaker (*β* = −0.03, *p* = 0.002). Identifying as a young person was associated with higher utilization of the Black female speaker (*β* = 0.05, *p* < 0.001) and Latinx female speaker (*β* = 0.04, *p* = 0.001) and lower utilization of NL White male speaker (*β* = −0.06, *p* < 0.001). Identifying as college-educated was associated with higher utilization of the Black female speaker (*β* = 0.03, *p* = 0.004) and lower utilization of the NL White male speaker (*β* = −0.03, *p* = 0.003). Finally, identifying as female was associated with higher utilization of the NL White male speaker (*β* = 0.04, *p* < 0.001) and lower utilization of the Latinx female speaker (*β* = −0.06, *p* < 0.001).

**Table 5:** Associations between meditation speaker proportions and demographics.

	NL white male speaker		Latinx female speaker		Asian male speaker		Black female speaker	
Demographic variable	*β*	*p*	*β*	*p*	*β*	*p*	*β*	*p*
Age (18–34)	−0.06	<0.001	0.04	0.001	0.02	0.029	0.05	<0.001
College	−0.03	0.003	0.01	0.369	0.02	0.023	0.03	0.004
Female	0.04	<0.001	−0.06	<0.001	−0.01	0.534	0.01	0.258
Married or domestic partnership	0.01	0.616	0.00	0.947	−0.01	0.275	0.00	0.787
Race African American	−0.02	0.132	0.00	0.941	−0.02	0.104	0.06	<0.001
Race Asian	0.03	0.012	−0.01	0.179	−0.01	0.503	−0.03	0.002
Race Latinx	0.01	0.536	0.02	0.065	−0.02	0.108	−0.02	0.015
Race Native American or Pacific Islander	0.00	0.706	0.00	0.990	0.00	0.844	0.01	0.520
Race other	−0.01	0.558	0.01	0.555	0.01	0.543	0.00	0.763

*NL* *White* Non-Latinx White, *β* standardized Beta, *Age* (<34), non-college educated, unmarried/divorced and NL White used as the reference group. *n* = 10,737. Statistical test: multiple regression.

As a sensitivity analysis, we examined whether associations between demographics and practice speaker proportions were consistent when limiting our examination to the first seven days of app utilization as opposed to the first 30 days (Supplementary Table 1). Six changes in statistical significance emerged. Associations between identifying as Asian, college educated, and as a young adult were no longer significantly associated with Black female speaker utilization. Additionally, identifying as Asian or college educated was no longer associated with utilization of the White male speaker. Conversely, identifying as Latinx was associated higher utilization of the Latinx female speaker (*β* = 0.03, *p* = 0.008).

We also assessed if the results were consistent when limiting the data to individuals who engaged in at least two practices in the core modules which would have allowed the opportunity to utilize speakers of two different racial/ethnic identities (Supplementary Table 2). Four changes in statistical significance emerged. Identifying as college-educated was no longer associated with utilization of the Black female speaker or NL White male speaker. Additionally, identifying as Asian was no longer associated with higher utilization of the White male speaker. Identifying as a young person was associated higher utilization of the Asian male speaker (*β* = 0.03, *p* = 0.009).

Finally, we assessed if the number of meditation practice sessions utilized by the participants varied as a function of the interaction between demographic variables and speaker portions (Supplementary Table 3). We conducted a large number of exploratory tests to examine this question. Of the 36 interaction tests between speaker proportions and demographics, only one emerged as significant after correcting for multiple comparisons. Specifically, Asian-identifying individuals who engaged at a higher proportion with the Black female speaker were more likely to utilize a higher number of meditation practice sessions (*B* = 13.00, *p* = 0.012). Results were consistent when limiting the data to individuals who engaged in at least two practices in the core modules. However, the association was non-significant when limiting the data to the first seven days of app utilization (*B* = 2.69, *p* = 0.091).

## Discussion

The present study examined if a freely available meditation app might reduce disparities in access and utilization of meditation in the US. Cost is considered to be a critical factor in reducing disparities in access to mental health apps^24^. However, our findings suggest that similar to findings in population-based surveys of meditation utilization^10,20,40^, certain groups, such as more highly educated and female individuals were more likely to access meditation through a freely available app whereas racial/ethnic minoritized groups, particularly African American and Latinx individuals, were less likely. We found that in addition to access, utilization also varied across demographic groups. Most notably, more highly educated individuals were more likely to utilize the app whereas other groups including African American and Native American/Pacific Islander individuals showed lower utilization. These findings are important because they bolster prior suggestions that cost alone is not a sufficient criterion for reducing disparities in access and sustained use of mobile health technologies^24^. Barring additional efforts to understand and reduce the basis for disparities in utilization, freely available and evidence-based technological interventions, such as HMP, have the potential to actually increase rather than decrease disparities in access, producing intervention-generated inequity whereby privileged groups receive disproportionate benefits^41^.

College education emerged as an important factor influencing both access and utilization of HMP. Nearly twice as many college-educated individuals accessed HMP relative to the general population and college education had the largest effect sizes when predicting utilization of the app (*βs* = 0.11–17). Several interrelated factors may be contributing to this phenomenon. First, education may serve as a proxy for income, a demographic data point that was not collected. Prior research suggests that nearly one in four adults in households with incomes less than $30,000 do not own a smartphone^42^. Thus, many individuals may simply be unable to access even freely available interventions requiring a smartphone. Second, prior research has also found that greater educational attainment is associated with higher levels of mental health awareness^43^, mobile health literacy^44^, mental health treatment seeking^45^, and general health consciousness^46^ which may lead to a higher proportion of college-educated individuals accessing and utilizing HMP. Higher levels of education have been found to be an important correlate of health^47^ and a lower education may be associated with less use of mental health services due to lower awareness of the potential benefits^45^. Furthermore, HMP focuses extensively on the science of meditation which may be more appealing to college-educated individuals, thus leading to greater access and utilization^48^. Finally, prior studies have found that individuals with marginalized identities experienced frustration and lack of confidence when navigating commercial mobile health apps which may hamper their ability to sustain utilization once they have accessed the app^49^.

Racial/ethnic identity also appears to be important in determining access and utilization. Specifically, African American and Latinx identities were associated with significantly lower access relative to representation in the general US population. This finding is consistent with disparities seen in access to mental health care^50^. Additionally, identifying as African American or Native American/Pacific Islander was also associated with lower utilization across all practice variables, albeit with very modest magnitude effect sizes (*β*s = −0.01 to −0.03). This finding aligns with prior research on mental health apps which also found lower utilization amongst minoritized groups relative to NL White individuals^51^. One potential reason for lower engagement with HMP might be that MBIs as currently developed in the US context are closely aligned with NL White cultural and social perspectives and may not be congruent with the day-to-day experiences of racial and ethnic minorities^15,18,24,52^. Recent meta-analyses have found that mindfulness and meditation-related research has lacked focus on diversity^53^ and used predominantly NL White samples^54^. Additionally, the connection of mindfulness with Buddhism may also be perceived as foreign and less appealing to racial/ethnic minoritized individuals given that some demographic groups (e.g., African Americans and Latinx) are more likely than NL Whites to have a strong influence of Christianity in their upbringing^18,55^.

One pathway to increase engagement amongst racial/ethnic minoritized individuals explored in the app was to allow users to choose among meditation practice speakers of various racial and ethnic backgrounds. App content was similar across all speakers and the NL White speaker was the primary guide for most didactic components and the default option for meditation practice. We found that relative to NL White individuals, African Americans chose the Black female speaker at a higher average proportion (*β* = 0.06), though the increased proportional utilization was not associated with increased overall use. Moreover, no significant associations were found between Latinx or Asian identities and the use of Latinx Female and Asian Male speakers, respectively. These findings align with prior psychotherapy research indicating that African Americans, more so than other groups, tend to prefer providers who match their racial or ethnic identity, though the effect of matching on treatment outcomes was small^26,27^. However, the lack of change in the association between demographics and meditation practice sessions suggests that minor changes in diverse representation by themselves may not be enough to increase utilization. Greater emphasis on diverse representation in the app as well as the use of modified and culturally congruent content may also be necessary to encourage greater use^25^. Finally, it should be noted that Asian identity was not significantly associated with variability in access relative to the general population and was largely not associated with variability in utilization. This may be due to the resonance between practices delivered in HMP and Asian cultural traditions^56^.

With regard to other demographic categories, results indicated that while female individuals were more likely to access HMP, they showed lower rates of utilization than non-females. Similarly, young people (18–34) were more likely to access the app relative to their proportion of the general population but had a negative association with the number of meditation practice sessions, although not days of app utilization or likelihood of engaging in any meditation practice. One potential reason for higher rates of accessing HMP is that women and younger individuals have been found to be more vulnerable to experiencing some common mental health challenges such as depression^57,58^ and may, for this reason, be more likely to access HMP to seek tools to support their mental health. Female individuals have also been shown to demonstrate more positive attitudes toward seeking mental health care and lower levels of stigma relative to males^59^. The age effect is almost certainly also influenced by the fact that younger people have greater comfort with technology^60^ and for this reason may be more likely to access mHealth at generally higher rates than older individuals^61^. Further research is necessary to understand potential causes of variation by gender and age.

The present study has important implications for research and inclusion, specifically digital inclusion in the development of mental health technologies. Digital inclusion has been defined as working to create equitable access to information and communication technologies for all individuals and communities, including those less privileged^62^. Results from the present study suggest that to achieve digital inclusion, app developers and evaluators may want to consider DEI factors beyond financial accessibility and diverse representation. Factors such as technological access and technological literacy as well as culturally appropriate content may be important to consider in the app design and marketing processes to achieve greater access and utilization of evidence-based mobile interventions^24,25,63^. Additionally, racial/ethnic minoritized users may have unique privacy concerns which may be important to address in the app development process^63^. Finally, it should also be noted that similar to other research on other mental health apps^64^, participant attrition was fairly high in the present study, with the average user utilizing the app for approximately four days and engaging in less than four meditation practice sessions. Such limited utilization may be unlikely to promote meaningful change for the average individual who downloaded the app. Additional efforts are needed to better understand how to overcome barriers to promote greater utilization of evidence-based digital tools^63^. At the same time, previous work has shown that modest amounts of engagement with a meditation app (e.g., average of 5 min of utilization per day over the course of four weeks)^31^ or even a single-session intervention^65^ can produce demonstrable change. Future research is warranted to identify which of the aforementioned factors are most critical to consider in app development both for increasing access and utilization as well as maximizing clinical effects. Greater participatory engagement with communities and individuals with marginalized identities in the design and development process may be crucial for achieving digital equity and inclusion^49^.

This study has several important limitations. First, the present study reports data from only one app. It is not possible to generalize to all mental health or meditation apps, although limited extant evidence suggests that access and utilization of more popular meditation apps may be skewed towards more privileged groups^21,22^. Second, the effect sizes were very small for most associations between demographics and utilization variables (*β*s < 0.10; Cohen^38^) suggesting that other factors may play a greater role in participant app utilization than presently included demographic factors. Nonetheless, a small magnitude effect can have meaningful cumulative effects^66^, especially for interventions such as self-guided mental health apps that can be readily delivered at scale. Additionally, the large sample size and general consistency across sensitivity analyses provide greater confidence in our results. Third, given that participants had a choice of whether to report demographic data, patterns of missingness (e.g., data missing not at random^67^) may have biased the results. Importantly, utilization data were never missing as utilization was collected passively through the HMP app. Fourth, while the present study attempted to examine the potential role of increased representation, much of the initial content is led by an NL White male speaker. Given, the rapid attrition found across mental health apps^64^, it is possible that many users were never exposed to practice speakers with diverse identities. Fifth, due to the nature of the data collection, age had to be dichotomized. This likely led to a loss of statistical power in analyses^68^. Finally, it should be noted that unmeasured confounding factors such as mental health-related stigma as well as a lack of awareness of meditation, meditation apps, and the associated potential health benefits might play a role in the demographic variability in access to HMP.

Several future directions follow from the current findings. First, it is vital to clarify the reasons driving lower access and utilization of HMP amongst non-college educated and racial/ethnic minoritized groups and to evaluate potential solutions for reducing this disparity. Specifically, examining the role of access and technological literacy as well as attitudinal and cultural factors which may impact implementation may be helpful^63^. It would be helpful to investigate factors such as mental health literacy, stigma, and beliefs regarding the likelihood that meditation may produce health benefits that may mediate associations between demographics factors and both access and utilization. Qualitative studies of non-meditators as well as individuals who have tried and discontinued their use of meditation apps may be particularly illuminating. Additionally, it would be helpful to utilize community-based participatory methods to explore a range of adaptations to understand which combination of factors (e.g., cost, content, representation, functionality) might lead to greater utilization of digital health interventions, particularly among racial/ethnic minoritized users. It may be helpful to test versions of the HMP with diverse speakers leading the introductory and didactic components of the app in addition to guided meditation practices. Second, it may also be valuable to compare the experience of participants with marginalized identities using different meditation apps, including comparisons with apps specifically designed for a cultural subgroup (e.g., Liberate^69^). Such comparisons could clarify what features or factors are most likely to drive access and utilization. Finally, an examination of demographic representation along with demographic predictors of access and utilization across other free and paid meditation apps would help extend the findings of the present study. Despite having a lower representation of certain demographic groups among HMP users relative to the general population (i.e., census proportions), HMP may include features (e.g., cost) that make it more accessible than other meditation apps. Although rates of some marginalized demographic groups (e.g., African Americans) were numerically higher in the current study relative to studies on other meditation apps (e.g., 5.3% vs. 1.4%^21^), study design features (e.g., the current study including all users who provided demographic data vs. previous studies including a convenience sample of users who consented to research) make direct comparison impossible.

Our findings suggest that disparities in meditation use and mental health care utilization found in the general population^10,20,40,50^, seem to extend to the digital realm. Despite being freely available, college-educated individuals accessed and utilized the HMP app whereas African Americans accessed and utilized the app at lower rates. Furthermore, although HMP includes speakers of diverse backgrounds and this content was more frequently accessed by some racial/ethnic minoritized individuals, this factor alone does not appear to increase utilization. Further efforts are needed to understand how to engage marginalized and minoritized groups with scalable evidence-based interventions.

### Supplementary information

Supplementary Materials Tables

## Data Availability

The datasets generated and analyzed during the current study are available from the corresponding author on reasonable request.

## Code availability

The code used to analyze the data is available from the corresponding author upon reasonable request.
