# Supplementary material for: Examining equity in access and utilization of a freely available meditation app
Source: Npj Ment Health Res. 2023 Apr 18;2:5. doi: 10.1038/s44184-023-00025-y (PMC10164442; doi:10.1038/s44184-023-00025-y)
Supplement: Supplementary file 1 — Supplementary Materials Tables [file 44184_2023_25_MOESM1_ESM.docx]

**Supplemental Materials: Examining Equity in Access and Utilization of a Freely Available Meditation App**

**Supplementary Materials Table 1**

Associations between Meditation Speaker Proportions and Demographics when Limiting to First Seven Days of HMP Use

|  | NL White Male Speaker | | Latinx Female Speaker | | Asian Male Speaker | | Black Female Speaker | |
| --- | --- | --- | --- | --- | --- | --- | --- | --- |
| Demographic Variable | *β* | *p* | *β* | *p* | *β* | *p* | *β* | *p* |
| Age (18-34) | -.05 | .001 | .04 | .006 | .02 | .208 | .03 | .018 |
| College | -.02 | .104 | .01 | .587 | .02 | .230 | .02 | .125 |
| Female | .04 | .002 | -.07 | .000 | -.02 | .203 | .02 | .063 |
| Married or Domestic Partnership | .00 | .946 | -.01 | .723 | .00 | .773 | .00 | .965 |
| Race African American | -.03 | .025 | .00 | .815 | -.01 | .698 | .08 | .000 |
| Race Asian | .01 | .283 | -.01 | .624 | -.02 | .105 | .00 | .878 |
| Race Latinx | .00 | .797 | .03 | .008 | -.02 | .082 | -.02 | .159 |
| Race Native American or Pacific Islander | .00 | .869 | -.01 | .433 | .00 | .821 | .01 | .587 |
| Race Other | -.01 | .591 | .02 | .184 | -.01 | .360 | .00 | .769 |

*Note.* HMP = Healthy Minds Program; NL White = Non-Latinx White. *β* = standardized Beta; Age (<34), non-college-educated, unmarried/divorced and NL White used as the reference group; Outcome = Sum Practice (number of meditation practice sessions); *n* = 10,737. Statistical Test: Multiple Regression

**Supplementary Materials Table 2**

Associations between Meditation Speaker Proportions and Demographics when Excluding all Individuals with One Meditation Practice or Fewer

|  | NL White Male Speaker | | Latinx Female Speaker | | Asian Male Speaker | | Black Female Speaker | |
| --- | --- | --- | --- | --- | --- | --- | --- | --- |
| Demographic Variable | *β* | *p* | *β* | *p* | *β* | *p* | *β* | *p* |
| Age (18-34) | -.06 | < .001 | .04 | .004 | .03 | .009 | .05 | < .001 |
| College | -.02 | .032 | .00 | .733 | .02 | .082 | .03 | .019 |
| Female | .03 | .003 | -.06 | .000 | .00 | .975 | .02 | .101 |
| Married or Domestic Partnership | .01 | .662 | .00 | .880 | -.01 | .437 | .00 | .859 |
| Race African American | -.02 | .043 | .00 | .906 | -.02 | .161 | .07 | .000 |
| Race Asian | .03 | .014 | -.01 | .329 | -.01 | .333 | -.03 | .002 |
| Race Latinx | .00 | .989 | .03 | .013 | -.01 | .195 | -.03 | .026 |
| Race Native American or Pacific Islander | -.01 | .455 | .00 | .756 | .00 | .697 | .01 | .429 |
| Race Other | -.01 | .585 | .01 | .517 | .00 | .749 | .00 | .904 |

*Note.* NL White = Non-Latinx White; *β* = standardized Beta; Age (<34), non-college-educated, unmarried/divorced, and NL White used as the reference group; Outcome = Sum Practice (number of meditation practice sessions). Statistical Test: Multiple Regression

**Supplementary Materials Table 3**

Interactions of Demographics and Speaker Proportions Predicting Meditation Practice

|  | NL White Male Speaker | | Latinx Female Speaker | | Asian Male Speaker | | Black Female Speaker | |
| --- | --- | --- | --- | --- | --- | --- | --- | --- |
| Demographic Variable | *B* | *p* | *B* | *p* | *B* | *p* | *B* | *p* |
| Speaker Proportion | -4.26 | < .001 | 3.10 | .015 | 5.04 | .003 | 8.93 | < .001 |
| Age (18-34) | -0.53 | .367 | -0.54 | .001 | -0.47 | .002 | -0.46 | .003 |
| College | 0.46 | .472 | 0.58 | < .001 | 0.62 | < .001 | 0.62 | < .001 |
| Female | 0.05 | .919 | -0.40 | .003 | -0.50 | < .001 | -0.50 | < .001 |
| Married or Domestic Partnership | 0.41 | .480 | -0.08 | .572 | -0.05 | .735 | -0.04 | .762 |
| Race African American | 0.30 | .810 | -0.69 | .047 | -0.57 | .095 | -0.70 | .041 |
| Race Latinx | 1.38 | .182 | -0.19 | .460 | -0.07 | .793 | -0.04 | .870 |
| Race Asian | -0.05 | .967 | 0.38 | .171 | 0.49 | .075 | 0.27 | .318 |
| Race Native American or Pacific Islander | 1.02 | .721 | -1.54 | .059 | -1.66 | .038 | -1.69 | .035 |
| Race Other | 1.49 | .314 | -0.60 | .148 | -0.40 | .331 | -0.41 | .305 |
| Age (18-34) x Speaker Prop | -0.06 | .923 | 0.47 | .616 | -0.39 | .760 | -2.58 | .099 |
| College x Speaker Prop | 0.11 | .879 | 0.82 | .416 | -0.54 | .693 | -1.75 | .316 |
| Female x Speaker Prop | -0.50 | .391 | 0.31 | .722 | 1.13 | .322 | 0.96 | .531 |
| Married or Domestic Partnership x Speaker Prop | -0.48 | .443 | 0.97 | .290 | 0.85 | .491 | 0.12 | .938 |
| Race African American x Speaker Prop | -1.11 | .419 | 1.92 | .369 | 1.09 | .770 | -2.26 | .313 |
| Race Latinx x Speaker Prop | -1.56 | .164 | 1.53 | .300 | 2.12 | .384 | 3.62 | .362 |
| Race Asian x Speaker Prop | 0.57 | .670 | 0.25 | .899 | -3.92 | 0.089 | 13.00 | .012 |
| Race Native American or Pacific Islander x Speaker Prop | -2.91 | .347 | 0.11 | .983 | 3.72 | .463 | 4.87 | .479 |
| Race Other x Speaker Prop | -2.17 | .177 | 3.16 | .155 | -0.65 | .824 | 2.17 | .616 |

*Note.* Speaker Prop = Speaker Proportion; NL White = Non-Latinx White; *B* = Beta; Age (<34), non-college-educated, unmarried/divorced, and NL White used as the reference group. Outcome = Sum Practice (in practice sessions); *n* = 10,737. Statistical Test: Multiple Regression
